# Upper Blepharoplasty for Dermatochalasis With or Without Resection of the Orbicularis Oculi Muscle, Preaponeurotic and Nasal Fat Pads: A Comparative Study

**DOI:** 10.1007/s00266-025-04657-7

**Published:** 2025-01-24

**Authors:** Juan A. Viscardi, Salvatore Giordano

**Affiliations:** Department of Plastic and General Surgery, Turku University Hospital and The University of Turku, Turku, Finland

**Keywords:** Dermatochalasis, Blepharoplasty, Nasal fat pad, Preaponeurotic fat pad, Orbicularis oculi muscle, Complications

## Abstract

**Objective:**

Upper blepharoplasty is the gold standard procedure for upper eyelid dermatochalasis. Upper blepharoplasties procedures include removing the skin, orbicularis oculi muscle, preaponeurotic, or nasal fat pad. The purpose of this study is to report surgical outcomes and compare them to the most common techniques.

**Material and Methods:**

A retrospective review of 386 consecutive patients who underwent upper blepharoplasty at Turku University Hospital from January 1st, 2015 to June 30th, 2017 was conducted. Data collected include patient demographics, surgical details, and details regarding the type and frequency of complications.

**Results:**

During the study period, 51 upper blepharoplasties with orbicularis oculi muscle excision, skin, preaponeurotic and nasal fat pads removal (study group) and 335 upper blepharoplasties with skin only removal (control group) were performed. Non-parametric tests showed that operative time (M=60.2min; SD=11.7min) and return to work (M=8.0days; SD=3.1days) were significantly shorter in the control group. No significant differences in the total amount of complications were detected (7.8% vs 2.4%, *p*=0.075). Subjective patients' satisfaction was significantly higher in the study group (from 0-10, mean 8.3 vs 7.0, *p*=0.034).

**Conclusions:**

When compared to skin-only blepharoplasty, upper blepharoplasty with orbicularis oculi muscle excision, removal of skin, preaponeurotic and nasal fat pad appears to be a safe surgery with improved patient satisfaction and without carrying on additional complications.

**Level of Evidence III:**

This journal requires that authors assign a level of evidence to each article. For a full description of these Evidence-Based Medicine ratings, please refer to the Table of Contents or the online Instructions to Authors www.springer.com/00266.

## Introduction

Dermatochalasis is a condition characterized by an excess of skin on either the upper or lower eyelid. Significant dermatochalasis of the upper lids can lead to lateral hooding and visual obstruction, while severe dermatochalasis of the lower lids may result in ectropion. Additionally, prolapse of the lacrimal glands or fat herniation can exacerbate these symptoms. With the visual field limitation, dermatochalasis might result in a persistent tension-type headache due to the restriction of the visual field [1–3]. Additionally, symptoms of dry eyes have been associated with this condition [4–7]. Objective ways to measure the visual field loss caused by dermatochalasis are perimeter tests, the measurement of the distance between lower border of the skin fold in the upper eyelid and the margin to reflex distance (MRD 1) defined as the distance between the upper lid margin and the corneal light reflex [8].

Blepharoplasty is the golden standard procedure to treat dermatochalasis and it is one of the most common plastic surgeries in the USA with over 200,000 operations annually [9]. Blepharoplasty alleviates visual field limit both subjectively and objectively [5]. Furthermore, it may lessen the symptoms of dry eyes and chronic headaches, enhancing quality of life and self-esteem [1, 2, 4, 7]. Upper blepharoplasty can result in significant improvement in patient happiness, self-consciousness of appearance, and benefit in everyday life [10–12].

Blepharoplasty is a procedure where different amounts and types of tissue are removed from the upper eyelid. The two most common approaches are to remove only the skin, or concomitant orbitalis oculi muscle and preaponeurotic, or nasal fat pad. However, complications can lead to poor functional and aesthetic outcomes. These include periorbital hematoma, surgical site infections, suture granulomas, lagophthalmos and post-operative ptosis [13–18]. Visual loss caused by ischemia or intraorbital hemorrhage is a rare complication which requires immediate treatment [14, 15, 18–20]. However, it is uncertain which approach is the safest, with the least complications and re-operations at long-term follow-up [12, 21, 22]. In Finland blepharoplasty is indicated in public health care settings when dermatochalasis causes functional deficits.

The aim of our study is to compare skin-only excision blepharoplasty to blepharoplasty involving concomitant removal of the orbicularis oculi muscle and preaponeurotic or nasal fat pads, performed in a reconstructive setting within a Finnish patient population of Western descent. Our hypothesis is that concomitant removal of orbicularis oculi muscle and preaponeurotic, or nasal fat pad removal is equally safe compared to skin-only blepharoplasty and could lead to superior aesthetic outcome regarding upper blepharoplasty.

## Material and Methods

### Study Design

This retrospective cohort study was approved by the Turku University Hospital District and conducted in accordance with the principles of the Declaration of Helsinki. Since the study was retrospective, ethical committee approval was not required. The study population was identified through electronic medical records.

### Study Population

The inclusion criteria for this study were a diagnosis of dermatochalasis and undergoing bilateral upper blepharoplasty, either skin-only or with removal of the orbicularis oculi muscle and preaponeurotic or nasal fat pads. Patients with a history of unilateral blepharoplasty or previous facial surgery were excluded. The diagnosis of dermatochalasis in a reconstructive setting required at least one of the following criteria: excess upper eyelid skin in direct contact with the eyelashes or causing the eyelashes to contact the eyeball, a margin reflex distance of less than 2 mm, or folding of the excess skin leading to inflammation and wounds in the eyelid area.

Patients who underwent surgery at Turku University Hospital between 2015 and 2017 were included. A total of 404 consecutive cases were identified, with 386 meeting the inclusion criteria. Eighteen patients were excluded due to unilateral blepharoplasty or prior periorbital surgery. The patients were divided into two groups: study and control. The study group included 51 individuals with dermatochalasis who underwent blepharoplasty with removal of the skin, orbicularis oculi muscle, and preaponeurotic or nasal fat pads. The control group consisted of 335 individuals with dermatochalasis who had skin-only blepharoplasty.

### Data Collection

The medical records of these patients were reviewed manually to collect the following information: general patient demographics, preoperative conditions of the orbital region, medications that increase the risk of bleeding, and comorbidities. Data on the surgical technique, equipment used, and local anesthesia were also extracted. Additionally, postoperative complications, re-operations, follow-up duration, and length of sick leave were analyzed (Table 1). Patient and surgeon satisfaction with the postoperative results was assessed using a visual analogue scale (VAS) from 0 (not satisfied) to 10 (completely satisfied).

**Table 1:** Demographics of patients at time of study

	*Fat/Orbicularis Resection Group (n = 51)*	*Control Group (n = 335)*	*p-value*
Age (mean ± SD)	62.8 ± 9.3	67.7 ± 10.1	**0.001**
BMI	27.3 ± 4.3	27.6 ± 6.6	0.703
Any comorbidity	33 (64.7%)	205 (62.1%)	0.723
HTA	27 (52.9%)	185 (55.1%)	0.777
Diabetes	9 (17.6%)	50 (14.9%)	0.615
Hypercholesterolemia	14 (27.5%)	127 (37.8%)	0.153
Lung disease	7 (13.7%)	48 (14.3%)	0.915
Depression	10 (19.6%)	33 (9.8%)	**0.038**
Smokers	14 (27.5%)	45 (13.4%)	**0.010**
Warfarin	2 (3.9%)	26 (7.8%)	0.325
Aspirin	7 (13.7%)	59 (17.6%)	0.498
Omega-3	3 (6.1%)	21 (6.3 %)	0.952
Ptosis	2 (3.9%)	16 (4.8%)	0.787
Asymmetry	17 (33.3%)	98 (29.3%)	0.553

Bold values indicate statistically significant *p*-values

### Definitions

A hematoma requiring revision is a large periorbital hematoma that causes symptoms and pain to the patient. A retrobulbar hematoma is a condition where the hematoma compresses the neurovascular structures of the eye and requires immediate evacuation of the hematoma and ophthalmological treatment. Ecchymosis was defined as excessive blood in the subcutaneous tissue not requiring intervention, but uncomfortable for the patient.

Surgical site complications were limited to the operation area, including granuloma formation, suture abscesses, eye dryness and lagophthalmos.

Surgical site infections included conjunctivitis, keratitis, wound infection, orbital preseptal cellulitis or orbital cellulitis.

Systemic complications or infections included sepsis, organ failure and other major conditions that require treatment in a hospital.

Re-operations included re-blepharoplasties, removal of granulomas and suture abscesses, brow lifts after blepharoplasty to enhance its results, or any operation that requires local anesthesia after the initial operation.

Furthermore, each patient was verbally surveyed about their satisfaction using a Visual Analogue Scale (VAS) ranging from 1 (worst) to 10 (excellent) during the final postoperative follow-up. Surgeons also provided their own scores using the same scale.

### Surgical Technique

The surgeries were carried out by 34 different surgeons, 22 of whom were ophthalmologists and 12 of whom were plastic surgeons. The surgical site was marked, and local anesthetic was administered. The marked skin was excised, as well as a strip of orbicularis oculi muscle, preaponeurotic or nasal fat pad, or a combination of both, depending on the method used. The indication for each procedure was determined by the surgeon's preference. Similarly, absorbable, or non-absorbable thread was employed to close wounds.

### Statistical analysis

Parametric and nonparametric data were presented as mean ± standard deviation (SD). All statistical analyses were performed using SPSS software (version 29.0, SPSS Inc., Chicago, IL, USA). Group comparisons were conducted using the chi-square test or Fisher’s exact test, as appropriate. Continuous variables were analyzed using the t-test. A 95% confidence interval was applied, and a two-sided P value of 0.05 was considered statistically significant.

## Results

Patients in the study group were significantly younger than those in the control group (62.8 ± 9.3 years vs. 67.7 ± 10.1 years, *p* = 0.001) and had a higher prevalence of smoking (14 [27.5%] vs. 45 [13.4%], *p* = 0.010). The study group also had a significantly higher prevalence of depression compared to the control group (10 [19.6%] vs. 33 [9.8%], *p* = 0.038). No other significant demographic differences were observed between the groups. Additionally, there were no significant differences in preoperative ptosis or orbital asymmetry (Table 1).

**Fig. 1:**
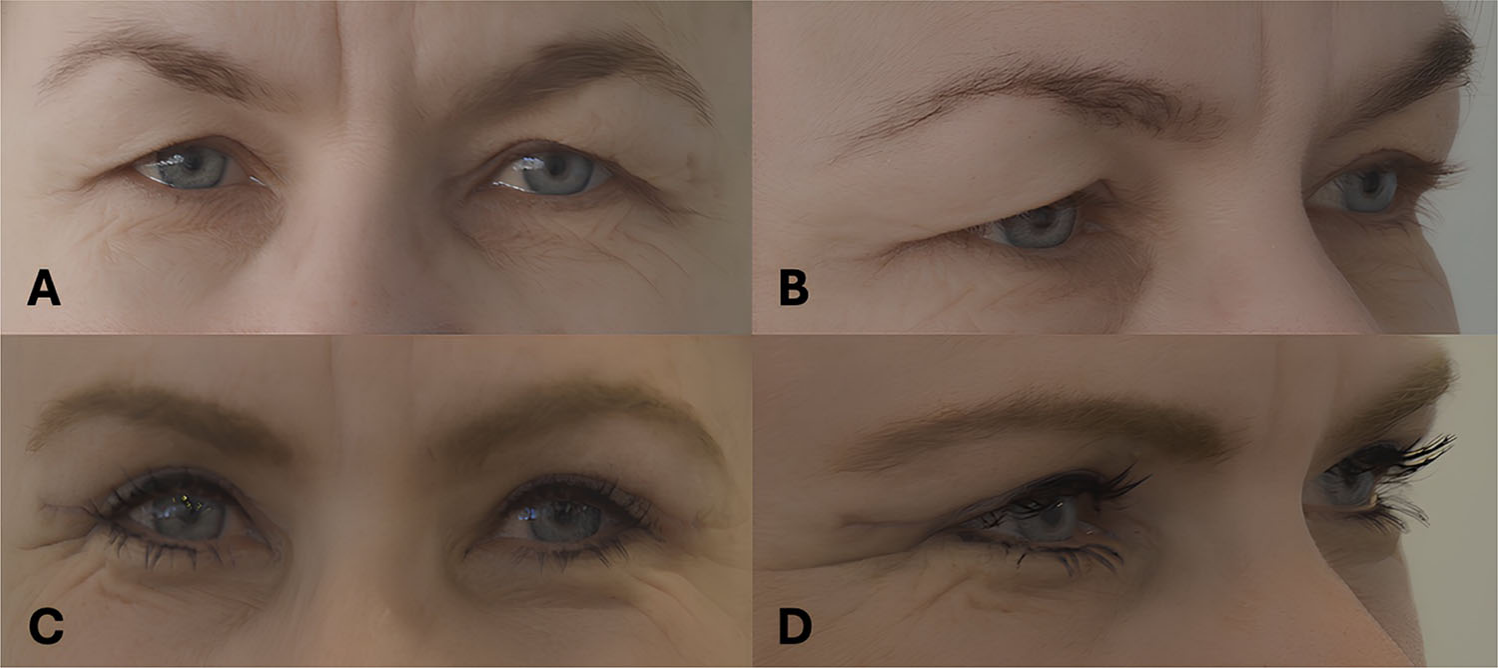
Preoperative view of a 55-year-old female patient (**A**, **B**), and 2 months after skin, orbicularis oculi muscle and preaponeurotic, or nasal fat pad removal blepharoplasty (**C**, **D**)

**Fig. 2:**
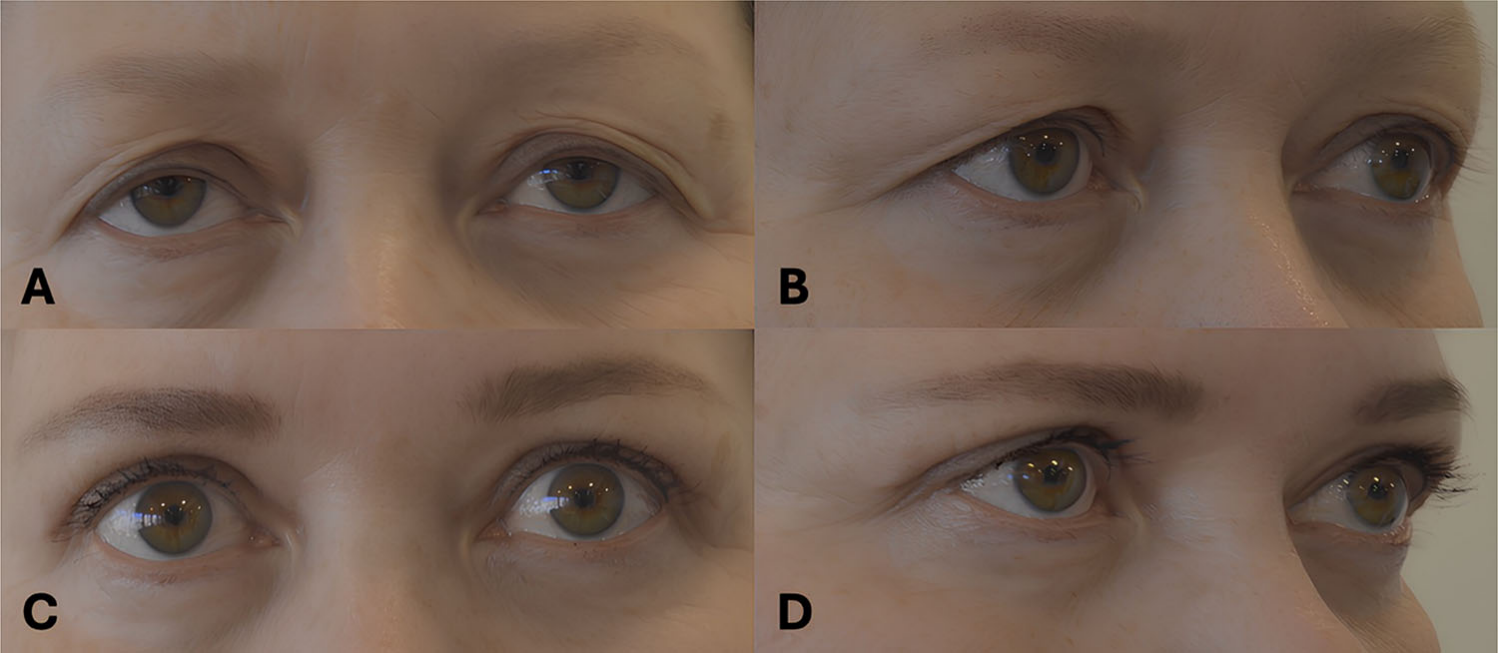
Preoperative view of a 58-year-old female patient (**A**, **B**), and 4 months after skin-only blepharoplasty (**C**, **D**)

Operative time was significantly longer in the study group (72.2 ± 22.8 minutes vs. 60.2 ± 11.7 minutes, *p* < 0.001), and the duration of sick leave was also significantly longer in the study group compared to the control group (11.3 ± 4.2 days vs. 8.0 ± 3.1 days, *p* < 0.001, Table 2). The incidence of ecchymosis was significantly higher in the study group (3.9% vs. 0.29%, *p* = 0.046). There were no significant differences in other complications between the groups. Although the re-operation rate was higher in the study group (11.8% vs. 7.7%), this difference was not statistically significant. Patients in the study group were significantly more satisfied with the postoperative results than those in the control group (VAS 8.3 ± 2.7 vs. 7.0 ± 2.3, *p* = 0.034). However, there were no significant differences in surgeons’ satisfaction (Table 3). Figure 1 presents a 55-years-old female patient before surgery (A,B), and 2 months after skin, orbicularis oculi muscle and preaponeurotic, or nasal fat pad removal blepharoplasty (C, D). Figure 2 presents a 58-year-old female patient before surgery (A, B), and 4 months after skin-only blepharoplasty (C, D).

**Table 2:** Comparison of perioperative parameters in the two groups of patients

	*Fat/Orbicularis Resection Group* *(n = 51)*	*Control Group (n = 335)*	*p-value*
Operative time (min, mean ± SD)	72.2 ± 22.8	60.2 ± 11.7	**<0.001**
Return to work (days, mean ± SD) *	11.3 ± 4.2	8.0 ± 3.1	**<0.001**
Follow-up (months, mean ± SD)	12.0 ± 10.3	15.6 ± 20.6	0.234

Bold values indicate statistically significant *p*-values

^*^For patients not retired (21 vs 73 patients).

**Table 3:** Postoperative complications and satisfaction

	*Fat/Orbicularis Resection Group*	*Control Group (n =335)*	P-value*
	*(n = 51)*		
Complications	3 (7.8%)	5 (2.4%)	0.075
Any infection	1 (0.3%)	3 (0.8%)	0.432
Ecchymosis (requiring any observation)	2 (3.9%)	1 (0.29%)	**0.046**
Postoperative ptosis	0 (0.0%)	1 (0.3%)	1.000
Levator damage	0 (0.0%)	0 (0.0%)	1.000
Re-operation	6 (11.8%)	26 (7.7%)	0.327
Patients’ satisfaction (0-10, mean ± SD)	8.3 ± 2.7	7.0 ± 2.3	**0.034**
Surgeon’s satisfaction (0-10, mean ± SD)	8.5 ± 2.4	7.4 ± 2.3	0.085

Bold values indicate statistically significant *p*-values

## Discussion

The present study aimed to evaluate the clinical outcomes of patients undergoing blepharoplasty with or without removal of the orbicularis oculi muscle, preaponeurotic fat, or nasal fat pad in a reconstructive context within a Western population treated at a university hospital. Our findings reveal several significant distinctions between the study group, who underwent more extensive procedures, and the control group, who received skin-only blepharoplasty.

Upper blepharoplasty is the gold standard procedure to treat dermatochalasis of the upper eyelid, and it also improves the aesthetic and psychological outcomes related to this condition [1, 2, 8, 12–14, 23–25].

A significant age difference between the study and control groups, with the study group being younger on average, emerged as one of the notable findings. This difference may indicate a selection bias, where younger patients are more likely to opt for extensive surgical procedures due to a greater emphasis on achieving improved cosmetic outcomes or due to age-related differences in skin and muscle characteristics. Additionally, the higher prevalence of smoking and depression in the study group suggests a different baseline health profile that could influence both the choice of surgical technique and postoperative recovery. Because smoking is a well-known cause of increase in postoperative complications, it may augment the incidence of complications [17, 26]. However, younger age in the study group may have played a role in reducing the total number of complications.

These results are corroborated by previous literature, which emphasized the importance of patient selection and correct information for possible preventive measures, such as abstaining from smoking at least four weeks before surgery [17, 24, 26]. On the other hand, surgeons might also have chosen a less invasive technique to correct dermatochalasis for elderly patients to avoid unnecessary risks. Advanced age and related comorbidities might pose the patient at an increased risk of bleeding and other surgical related complications.

The operative time was significantly longer in the study group, which is consistent with the more complex nature of the procedures involving muscle and fat removal. This extended operative time contributed to the significantly longer duration of sick leave observed in the study group. The longer recovery period may also be associated with the more invasive nature of the surgery, necessitating a longer healing process. However, a previous study on this topic showed that the patients return to work in similar time lapse, independently on the type of surgical technique and tissue resected. Almost 60% of the patients had returned to work after one week and 90% after two weeks [11]. The results of our study indicate that there is no reason to prescribe longer sick leaves for patients with a more invasive blepharoplasty technique, with a median length under two weeks for both groups in the case the patient was an active worker.

Minor complications occurred at a rate of 2.4 % in the control group and 7.8 % in the study group. No major complications occurred. Interestingly, the incidence of ecchymosis was significantly higher in the study group. This finding is consistent with the more extensive tissue manipulation required in procedures involving muscle and fat removal, which could lead to increased postoperative bruising. However, no significant differences were observed in other complications, suggesting that while more invasive procedures may increase certain risks, they do not necessarily lead to a broader range of postoperative complications.

Although the re-operation rate was higher in the study group, this difference did not reach statistical significance. This finding might indicate that while more complex procedures can lead to a slightly increased likelihood of requiring additional surgery, this risk is not markedly higher. Importantly, patients in the study group reported significantly higher satisfaction with the postoperative results compared to the control group. This suggests that despite the increased complexity, longer recovery, and higher risk of ecchymosis, patients who undergo more comprehensive procedures may perceive the outcomes as more favorable. Notably, surgeon satisfaction did not differ significantly between the two groups, indicating that the surgical outcomes were viewed similarly by professionals, regardless of the procedure's extent.

The optimal type and amount of tissue to be removed in blepharoplasty is still debated, but most of studies mainly focus either on the aesthetic or the functional outcomes [27]. Some authors claim that saving the orbital fat and orbitals oculi muscle preserves a youthful look while other studies have shown that the initial results might be worse after the removal of part of orbicularis, but the final aesthetic results are similar [1, 17, 22, 27, 28]. Our findings indicate that patients who underwent blepharoplasty with removal of the orbicularis oculi muscle and either the preaponeurotic or nasal fat pad reported higher satisfaction at follow-up compared to those who received skin-only blepharoplasty. However, this satisfaction may be influenced not only by the aesthetic outcome but also by functional improvements. In the study group, only minimal removal of the nasal or preaponeurotic fat pads was performed, underscoring the reconstructive rather than cosmetic intent of the procedure.

As expected, most postoperative complications were minor and treated conservatively and there were no statistical differences in the number of re-operations among the two groups. Most of the reoperations were due to dermatochalasis recurrence and removal of suture granulomas or abscesses. The study group had significantly more ecchymosis, which could be expected from a more invasive surgery. Interestingly, the only post-operative ptosis happened after skin-only blepharoplasty, which is considered to be a less invasive procedure [17, 29]. Other authors found that the number of complications depend mostly on the patients’ primary disease and not on the blepharoplasty technique itself [12, 22]. Some studies have detected that minor complications such as itching, pain, dry-eye symptoms, edema, hematoma and even lagophthalmos were worse when a part of orbicularis oculi muscle was removed particularly during the first postoperative weeks, but these differences were not persistent and resolved during the follow-up [15, 16]. In our study, we could not assess such minor complications due to lack of data.

It has been shown that a part of orbicularis oculi muscle, which is typically removed in blepharoplasty, acts as a tear pump [12]. Therefore, blepharoplasty can even exacerbate the dry eye symptoms if part of the orbicularis oculi muscle is removed without a careful assessment [4–6]. On the other hand, weakening the upper part of the orbicularis oculi might result in a mild upper brow lift, improving both functional and aesthetic outcomes.

Both blepharoplasty techniques seem to be safe, and patients seems to have a higher satisfaction in the study group. However, it seems that removing the excess fat or muscle does not significantly prevent the need for reoperation. Though, there are subgroups such as patients with lacrimal gland prolapse or fat herniation that might benefit from a more invasive surgery.

This study has several limitations. The interventions and patients were in a teaching hospital addressed to a reconstructive setting, not an aesthetic one. The retrospective nature of the study introduces potential biases, particularly in patient selection and data collection. The study population consisted exclusively of individuals of Western descent, whose skin aging characteristics, particularly in the periorbital region, differ from those of other ethnic groups, such as Latino, African American, East Asian, and South Asian populations [30–33]. Additionally, the lack of randomization between the study and control groups may confound the results, as patient characteristics likely influenced the choice of surgical technique. Similarly, the satisfaction of both patients and surgeons was assessed subjectively using a Visual Analog Scale (VAS) ranging from 0 to 10. However, patient dissatisfaction was not specifically analyzed with regard to individual anatomical elements. Furthermore, the follow-up period varied between patients, which could affect the assessment of long-term complications and satisfaction.

To our best knowledge this is one of the largest studies evaluating post-operative complications in patients with dermatochalasis after blepharoplasty. However, the small number of patients in the study group may explain wherefore the number of complications and re-operations did not achieve statistical significance. Further studies with larger study populations are warranted to clarify the indications for one procedure over the other.

## Conclusion

When compared to skin-only blepharoplasty, upper blepharoplasty with the excision of the orbicularis oculi muscle, skin, nasal, or preaponeurotic fat pad is a safe procedure with comparable results and no statistically significant difference regarding complications. Skin only blepharoplasty appears to be a faster procedure than blepharoplasty with orbicularis oculi muscle and preaponeurotic, or nasal fat pad removal. While more extensive blepharoplasty procedures involving muscle and fat removal are associated with longer operative times, increased incidence of ecchymosis, and longer recovery periods, they also result in higher patient satisfaction without significantly increasing the overall complication rate. These findings suggest that patient selection and preoperative counseling should consider both the risks and potential benefits of more invasive blepharoplasty techniques, particularly in younger patients with a higher baseline prevalence of smoking and depression. However, these patients tend to experience higher satisfaction after orbicularis oculi muscle and preaponeurotic, or nasal fat pad, are removed. Further prospective studies with standardized follow-up protocols are warranted to confirm these results and better guide surgical decision-making in blepharoplasty.
